# Adrenal and Gonadal Activity, Androgen Concentrations, and Adult Height Outcomes in Boys With Silver-Russell Syndrome

**DOI:** 10.3389/fendo.2019.00829

**Published:** 2019-12-10

**Authors:** Kjersti Kvernebo Sunnergren, Carina Ankarberg-Lindgren, Jovanna Dahlgren

**Affiliations:** ^1^Göteborg Pediatric Growth Research Center (GP-GRC), Department of Pediatrics, Institute of Clinical Sciences, Sahlgrenska Academy, University of Gothenburg, Gothenburg, Sweden; ^2^Department of Pediatrics, Ryhov County Hospital, Jönköping, Sweden; ^3^Department of Endocrinology, Sahlgrenska University Hospital, Queen Silva Hospital, Gothenburg, Sweden

**Keywords:** small for gestation age, androgens, hypogonadism, anti-Müllerian hormone, mass spectrometry, Silver-Russell syndrome, adult height, adrenarche

## Abstract

**Background:** We have previously shown that adult height (AH) in males with Silver-Russell syndrome (SRS) correlated negatively with prepubertal estradiol concentrations. We aimed to identify the source of estradiol by analyzing androgen secretion profiles and measuring anti-Müllerian hormone (AMH) and inhibin B concentrations during childhood and puberty in this group of patients.

**Methods:** In a retrospective longitudinal single-center study, 13 males with SRS were classified as non-responders (NRs = 8) or responders (Rs = 5), depending on the AH outcome. From 6 years of age, androgens were determined by mass spectrometry, and AMH, inhibin B and sex hormone-binding globulin concentrations were analyzed by immunoassays.

**Results:** AH outcome correlated negatively with dehydroepiandrosterone-sulfate (DHEAS) at 8 (*r* = −0.72), 10 (*r* = −0.79), and 12 years (*r* = −0.72); testosterone at 10 (*r* = −0.94), 12 (*r* = −0.70) and 14 years (*r* = −0.64); dihydrotestosterone (DHT) at 10 (*r* = −0.62) and 12 years; (*r* = −0.57) and AMH at 12 years (*r* = 0.62) of age. Compared with Rs, NRs had higher median concentrations of DHEAS (μmol/L) at 10 years (2.9 vs. 1.0); androstenedione (nmol/L) at 10 (1.1 vs. 0.6) and 12 years (1.7 vs. 0.8); testosterone (nmol/L) at 10 (0.3 vs. 0.1), 12 (7.8 vs. 0.2) and 14 years (15.6 vs. 10.4); and DHT (pmol/L) at 10 (122 vs. 28) and 12 years (652 vs. 59) of age. AMH (ng/mL) was lower in NRs than in Rs at 12 years of age (11 vs. 50). No significant differences were observed in the inhibin B concentrations at any age.

**Conclusions:** The elevated androgen concentrations before and during puberty, originated from both adrenal and gonadal secretion and correlated negatively with AH outcomes in males with SRS.

## Introduction

Silver-Russell syndrome (SRS) is a rare and heterogeneous syndrome affecting 1:30 000-100 000 births and is characterized by intrauterine growth restriction, short stature, relative macrocephaly with a prominent forehead, hemihypotrophy and a variety of minor malformations, including an increased risk of external genital malformations in boys (1–3). Epigenetic changes are found in some but not all individuals (1). Smeets et al. reported a similar total height gain from the start of growth hormone (GH) treatment in SRS patients compared to that in non-SRS patients born small for gestational age (SGA). Moreover, the height standard deviation score (SDS) declined in both groups during puberty, although a steeper decline and shorter adult height (AH) compared to mid-parental height (MPH) was observed in SRS patients (4). Hence, children with SRS have a better prepubertal height catch-up but an impaired pubertal growth spurt compared to non-SRS children born SGA. In a recent publication, we found a strong and negative correlation between estradiol (E_2_) levels at 10 years of age and AH height outcome in GH-treated males with SRS and we concluded that early E_2_ exposure affects skeletal maturation, resulting in impaired pubertal height gain, negatively affecting AH outcome (5). However, the source of E_2_ was not identified. Premature adrenarche is reported to be more frequent in children with SRS but does not seem to affect AH in this group of patients (1, 6). Small adult testicular volumes (5, 7) and Sertoli cell dysfunction (7) have been reported in males with SRS resulting in a risk of underestimating pubertal development when assessing testicular size and thereby, gonadal derived androgens might contribute to elevated androgen concentrations even at prepubertal testicular volumes.

The hypothesis of this study was that testicular activity rather than adrenal activity causes increased androgen secretion, leading to elevated E_2_ concentrations through aromatization in GH-treated males with SRS.

## Materials and Methods

### Study Population

The study population consisted of 13 males with SRS, all treated with GH from an early age. Eleven of the subjects have previously been described in detail including data on epigenetic testing as well as data related to birth size, GH treatment, bone age, gonadotropin concentrations and pubertal development (5). Depending on the AH outcome, defined as AH adjusted for MPH, subjects were retrospectively divided into one of two groups: eight subjects with AH >1 SDS below the MPH were defined as non-responders (NRs), and five subjects with AH ≤ 1 SDS below the MPH were defined as responders (Rs). The cohort was found to have epigenetic findings comparable to larger cohorts (1, 5). The two patients added to the cohort after reaching AH had both ≥4 Netchine-Harbison scores (8) and were born at full term. When tested for epigenetic changes in 11p15 and maternal uniparental disomy of chromosome 7, one patient demonstrated no epigenetic changes, and the other patient had epigenetic changes in 11p15. Neither of the two had clinical signs of pubarche or pubertal development before 10 years of age. One patient had pubarche at 10.1 years and testicular volume of 3 mL at 10.8 years of age. For the other patient, pubarche and the first testicular volume of ≥3 mL was recorded at 11.8 years of age. Both were classified as NRs. At AH, the patient with no epigenetic findings had a testicular volume of 15 mL. The other patient came for height measurements until he reached AH but did not undergo clinical examination with an assessment of testicular volume and did not leave blood samples after the age of 14 years. At the time, he had a testicular volume of 6 mL.

### Study Protocol

Height and weight were routinely recorded until AH. Pubertal onset was defined as a testicular volume of ≥3 mL (5), and AH was defined as a growth velocity of <1 cm/year (9). Blood samples were drawn in the morning (08:00–11:00 a.m.), initially and then yearly during GH treatment, following GH treatment protocols. After separation, sera were stored at −80°C. In this study, testicular volumes and blood samples were examined at ages 6, 8, 10, 12, 14, and 16 years with two NRs and one R lost to follow-up at 16 years. Androgens were also analyzed in early puberty if testicular volumes of 3–6 mL were not present at these ages. In addition to androgens, anti-Müllerian hormone (AMH) and inhibin B were analyzed every second year from 6 to 16 years. Sex hormone-binding globulin (SHBG) concentrations were obtained from patient records or, were analyzed retrospectively, although results were missing at 6 years of age in one NR and at 14 years of age in another NR. For two NRs, AMH and inhibin B were extrapolated at 6 years (using values at 8 years when the patient still had prepubertal levels) and 12 years of age (using the mean value of 10 and 14 years), respectively.

### Hormone Analyses

Serum dehyroepiandrosterone-sulfate (DHEAS) and androstenedione (A_4_) concentrations were simultaneously determined by liquid chromatography-tandem mass spectrometry from Agilent Technologies (Montréal, Canada). The lower limit of detection (LOD) was 0.1 μmol/L for DHEAS and 0.1 nmol/L for A_4_ (10). The total coefficient of variation (CV) was 4% at 1.1 μmol/L for DHEAS and 15% at 1.0 nmol/L for A_4_. Serum concentrations of testosterone (T) and dihydrotestosterone (DHT) were simultaneously determined by high-sensitive gas chromatography-tandem mass spectrometry (Agilent Technologies, Montréal, Canada). The LOD was 0.1 nmol/L for T and 27 pmol/L for DHT (11). The total CV for T was 16% at 0.3 nmol/L and 8% at 20 nmol/L; for DHT, the total CV was 20% at 70 pmol/L and 13% at 700 pmol/L (11). Previously reported puberty specific reference intervals were used for comparison (10, 11).

As a biochemical definition of adrenarche, we used DHEAS concentrations exceeding 1.4 μmol/L suggested by several authors (6, 12).

The free androgen index (FAI) was calculated as (T/SHBG) × 10^4^ and (DHT/SHBG) × 10^4^.

AMH and inhibin B were analyzed using enzyme-linked immunosorbent assay (ELISA) (AnshLabs, USA). The LOD was 0.023 ng/mL for AMH and 1.6 pg/mL for inhibin B. The total CV was <6% for AMH and <8% for inhibin B in the entire range. SHBG was determined using the electrochemiluminescence method with Cobas 8000 (Roche Diagnostics, Scandinavia AB). The LOD was 0.1 nmol/L and the total CV was 7% at a concentration of 40 nmol/L and 9% at 100 nmol/L.

### Statistical Analyses

Data are expressed as the median (range), unless stated otherwise. Hormone concentrations below LOD were set to LOD/2. Correlations between hormones at different ages and AH adjusted for MPH were calculated using Spearman's correlation coefficient. Statistical comparisons between groups were made at ages closest to 6.0, 8.0, 10.0, 12.0, 14.0, and 16.0 years using the Mann-Whitney *U*-test. IBM SPSS Software Corp. (version 25) (USA) was used for statistical analysis. Figures were drawn using Origin 9.0 (OriginLab Corp., Northampton, MA, USA). A *P* < 0.05 was considered significant.

## Results

### Correlations Between Hormones and AH Outcome

Significant correlations between the concentrations of gonadotropins, androgens, SHBG, FAIs, AMH, inhibin B at different ages and AH SDS adjusted for MPH SDS are shown in Table 1. DHEAS concentrations at 8, 10, and 12 years of age and T concentrations at 10, 12, and 14 years of age as well as DHT concentrations and FAIs for T at 10 and 12 years of age were negatively correlated with AH outcome, Table 1. At 14 years of age, FAIs for T had a borderline significant correlation with AH outcome in NRs (*r* = 0.57 and *P* = 0.051). Furthermore, AH outcome correlated positively with concentrations of AMH at 12 years of age. No significant correlations were found for gonadotropins, SHBG, FAIs for DHT or inhibin B at any point. At 6 and 16 years of age, no correlations were found for any hormone.

**Table 1:** Significant correlations between hormones at different ages and adult height (AH) outcome defined as AH SDS adjusted for mid-parental height SDS in 13 boys with Silver-Russell syndrome.

**Androgens (at diifferent ages in years)**	**Spearman's Rho**	***P*-values**
DHEAS (8)	−0.72	0.006
DHEAS (10)	−0.79	0.001
DHEAS (12)	−0.72	0.006
T (10)	−0.94	0.000
T (12)	−0.70	0.008
T (14)	−0.64	0.018
DHT (10)	−0.62	0.025
DHT (12)	−0.57	0.041
FAIT (10)	−0.75	0.003
FAIT (12)	−0.57	0.044
AMH (12)	0.62	0.025

*P < 0.05 were considered to represent statistically significant differences. AMH, anti-Müllerian hormone; DHEAS, dehydroepiandrosterone-sulfate; DHT, dihydrotestosterone; FAIT, free androgen index for testosterone; T, testosterone*.

### Hormones in Relation to Age

**Figure 1:**
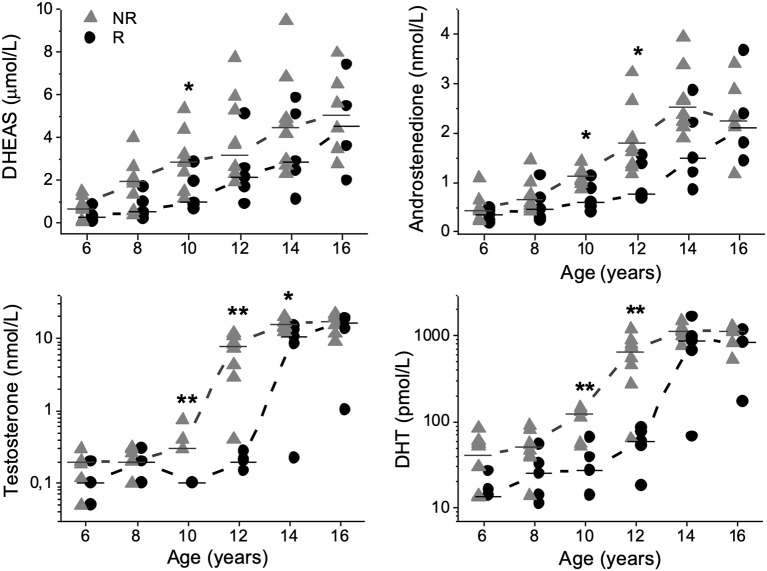
Dehydroepiandrosterone-sulfate (DHEAS), androstenedione, testosterone, and dihydrotestosterone (DHT) plotted relative to age in 13 boys with Silver-Russell syndrome. Median values were calculated and denoted as a line for each group and age. Triangles represent non-responders (NRs), and circles represent responders (Rs). *P < 0.05 and **P < 0.01.

Compared with Rs, NRs had higher concentrations of DHEAS (μmol/L) at 10 years [2.9 (1.1–5.4) vs. 1.0 (0.6–2.9); *P* = 0.040]; A_4_ (nmol/L) at 10 years [1.1 (0.9–1.4) vs. 0.6 (0.4–1.1); *P* = 0.023] and 12 years [1.7 (1.2–3.2) vs. 0.8 (0.7–1.5); *P* = 0.034] of age (Figure 1). At 10, 12, and 14 years of age, T (nmol/L) was higher in NRs than in Rs [0.3 (0.3–0.8) vs. 0.1 (0.1–0.1); *P* = 0.002], [7.8 (0.4–11.8) vs. 0.2 (0.1–0.3); *P* = 0.003] and [15.6 (12.2–20.2) vs. 10.4 (0.2–14.9); *P* = 0.028], respectively (Figure 1). Correspondingly, FAIs for T were higher in NRs than in Rs at 10 [59 (44–112) vs. 26 (10–38); *P* = 0.003], 12 [1 536 (91–4 609) vs. 43 (17–74); *P* = 0.003] and 14 years [6 325 (3 211–8 923) vs. 2 214 (52–5 707); *P* = 0.019] of age, respectively. Furthermore, DHT (pmol/L) was higher in NRs than in Rs at 10 and 12 years of age [122 (51–146) vs. 28 (<27–66); *P* = 0.008] and [652 (64–1 191) vs. 59 (<27–85); *P* = 0.008], respectively (Figure 1). Similarly, FAIs for DHT were higher in NRs than in Rs at 10 [23 (3–38) vs. 5 (1–13); *P* = 0.019] and 12 years of age [132 (15–331) vs. 16 (4–21); *P* = 0.013], respectively.

At 6 years of age, one NR had DHEAS concentrations consistent with adrenarche. At 8 years of age there were six NRs and one R and at 10 years of age seven NRs and two Rs with DHEAS concentrations exceeding the biochemical cut-off for adrenarche (6, 12).

**Figure 2:**
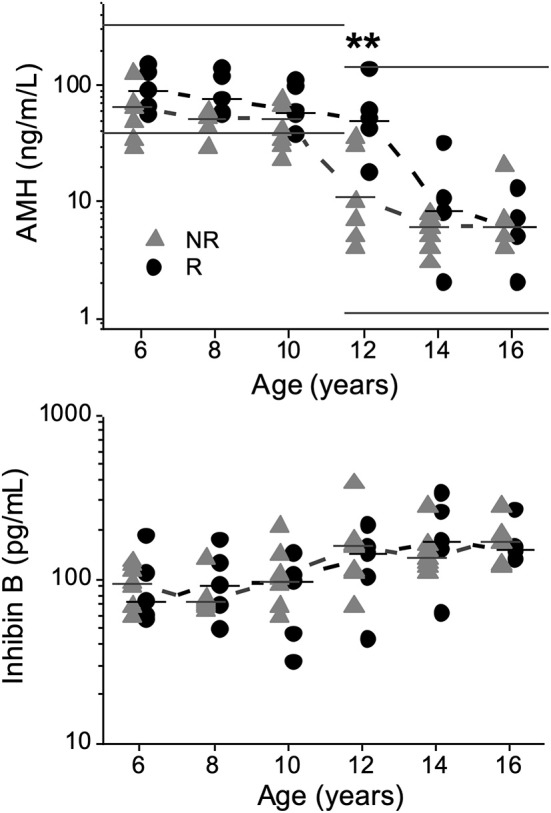
Anti-Müllerian hormone (AMH) and inhibin B concentrations plotted against age in 13 boys with Silver-Russell syndrome, showing a significant difference for AMH at 12 years of age (**P < 0.01). Median values were calculated and denoted as a line for each group and age. Normal reference intervals for AMH according to the manufacturer (AnshLabs, USA) at different ages are marked by straight lines. Triangles represent non-responders (NRs), and circles represent responders (Rs).

AMH concentrations (ng/mL) were significantly lower in NRs than in Rs at 12 years of age [11 (4–35) vs. 50 (18–134); *P* = 0.008], respectively (Figure 2).

No significant differences were observed in SHBG or inhibin B concentrations at any age (Figure 2). At 16 years of age, no significant differences were observed in the concentrations of any hormone; see Supplement.

### Hormones in Relation to Testicular Volume

**Figure 3:**
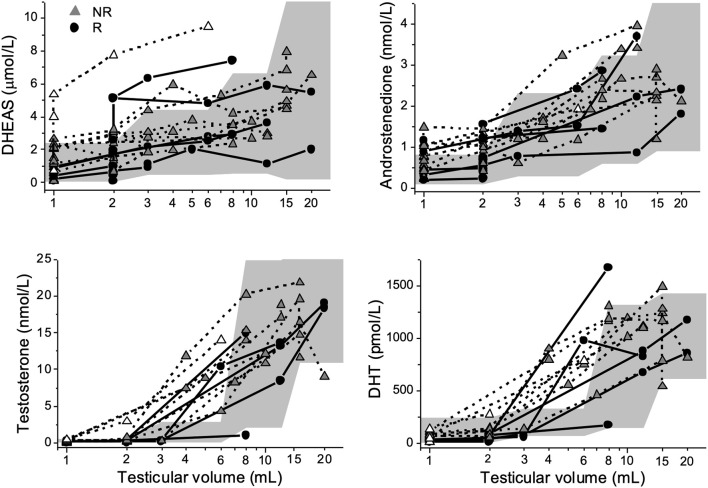
Dehydroepiandrosterone-sulfate (DHEAS), androstenedione, testosterone, and dihydrotestosterone (DHT) plotted against testicular volume in 13 boys with Silver-Russell syndrome. The gray area depicts reference intervals (2.5th-97.5th percentiles) for healthy controls (10, 11). Triangles represent non-responders (NRs), and circles represent responders (Rs). One NR was lost to follow up and had a testicular volume of 6 mL (marked with a white triangle).

Testicular volumes at ages 6–16 years for each patient are shown in Table 2. Several boys had elevated androgen concentrations prepubertally and in early pubertal stages compared to reference intervals (Figure 3). Before puberty, at testicular volumes of 1–2 mL, five NRs and two Rs were found to have elevated concentrations of DHEAS, and seven NRs and three Rs had elevated A_4_ concentrations. Moreover, two NRs showed elevated T concentrations, and one NR had elevated DHT compared to normal reference intervals. In early puberty, at a testicular volume of 3–6 mL, two NRs and two Rs had elevated DHEAS concentrations, and one NR and one R had elevated A_4_ concentrations compared to normal reference intervals, whereas five NRs and one R had concentrations of T and DHT above reference intervals (Figure 3). At 3–6 mL values on T and DHT were missing in two NRs and two Rs.

**Table 2:** Testicular volumes in 13 boys with Silver-Russell syndrome at ages 6, 8, 10, 12, 14 and 16 years.

**Patient number (R/NR)**	**Testicular volume (mL) at 6 years**	**Testicular volume (mL) at 8 years**	**Testicular volume (mL) at 10 years**	**Testicular volume (mL) at 12 years**	**Testicular volume (mL) at 14 years**	**Testicular volume (mL) at 16 years**
1 (R)	1	1	2	3	12	20
2 (R)	1	2	2	2	12	20
3 (NR)	2	2	2	10	15	20
4 (R)	2	2	2	2	8	Missing data
5 (NR)	1	1	2	3	10	Missing data
6 (NR)	1	1	2	6	15	15
7 (R)	1	1	1	3	6	12
8 (NR)	1	1	1	4	8	8
9 (NR)	1	1	2	5	12	12
10 (R)	1	2	2	2	2	8
11 (NR)	1	1	3	7	15	15
12 (NR)	1	1	1	4	6	Missing data
13 (NR)	2	2	2	4	8	15

*R, responder; NR, non-responder*.

## Discussion

In this retrospective study, we report significant negative correlations between androgen levels and AH outcome, namely, DHEAS at 8–12 years of age and T between 10 and 14 years of age as well as DHT and FAIs for T at 10–12 years of age. In support of these findings, concentrations of androgens were already higher from 10 years of age in NRs than in Rs. At 12 years of age, AMH was positively correlated with AH outcome, and lower AMH concentrations were found in NRs. AMH declined 2 years earlier in NRs compared to Rs, most likely due to higher concentrations of T in NRs. However, there were no differences in inhibin B concentrations at any age. This is the first systematic longitudinal study retrospectively examining androgen secretion patterns in GH-treated males with SRS, using mass spectrometry-based methods and identifying correlations between these hormones and AH outcome.

### Adrenal-Derived Androgens

DHEAS is one of the best serum markers of adrenal androgen secretion and thereby reflects the activity of the adrenal gland (13, 14). Premature adrenarche is a clinical entity defined by increased DHEAS concentrations followed by pubarche before age 9 years in boys (12). However, pubarche not only depends on the level of DHEAS but also reflects the androgen receptor sensitivity in hair follicle dermal papilla cells (15–17). A_4_ originates from both the adrenal and gonadal glands and may be converted to T and DHT in peripheral tissue (13, 18–22). Consequently, mildly increased concentrations of T and A_4_ are observed in children with premature adrenarche (23). In the present study, increased A_4_ concentrations were observed at testicular volumes of 1–2 mL in the majority of patients. Although no patient in this study met the definition of premature adrenarche, there were consistent and negative correlations between AH outcome and DHEAS at 8 to 12 years of age and several patients mostly NRs, had DHEAS concentrations corresponding to levels seen at adrenarche from 8 years of age. Compared with Rs, NRs had more pronounced DHEAS concentrations at 10 years of age, which, considering the lack of pubarche at this age, although speculative, may reflect partially impaired sensitivity of the androgen receptor. Binder et al. reported data on adrenarche in a cohort of 34 boys with SRS in which data on AH were available in 24 patients. The authors concluded that premature adrenarche is more common in patients with SRS, but AH outcomes seem not to be affected (6). These results are not consistent with the results of this study and might be due to the fact that Binder et al. seem to have included data on patients with only 3 Netchine-Harbison scores. Furthermore, the study design of this study with the subgrouping of patients according to AH outcome enabled us to identify differences in androgen secretion patterns within a group of males with SRS.

Altogether, this study suggests that males with SRS and pronounced adrenal activity may be at risk of impaired AH outcome, and adrenal derived androgens are likely to contribute to the observed elevated E_2_ concentrations (5).

### Gonadal-Derived Androgens

The gonads are reported to be the dominant source of T during male puberty, and in the adult male, <5% of T originates from A_4_ (21, 24). In this study, we found that five NRs and one R had T concentrations above reference intervals in early puberty. Our findings are not in line with a recent publication that reported prepubertal and pubertal T concentrations within reference intervals in 14 males with SRS (7). The diverse results might be due to inaccurate use of reference intervals established by immunoassay and not mass spectrometry in the previous publication, giving falsely high reference values. Considering the highly pronounced T and DHT concentrations found in NRs and the strong and negative correlations between these androgens and AH outcome, one might speculate that concentrations of A_4_, T and DHT already at 10 years of age at least partly originate from the gonads but further longitudinal studies on larger cohorts are needed to confirm the contribution of the gonads. However, all except for one NR lacked clinical signs of puberty, and all subjects had prepubertal concentrations of gonadotropins at 10 years of age (5). On the other hand, underestimating pubertal development based on testicular volumes is a risk in boys with SRS (5). Furthermore, Albertsson-Wikland et al. have previously reported a striking rise in LH levels, with a clear day-night rhythmicity, already at testicular volume of 3 mL, suggesting activation of the HPG-axis already at this point (25). In this cohort gonadotropins were analyzed in the morning (5) and therefore the finding of low gonadotropins in this cohort does not exclude activation of the HPG-axis.

### Gonadal Function

At prepubertal stages, testicular volume mostly reflects Sertoli cell mass, but during puberty, germ cells, which are dependent on factors secreted by Sertoli cells, contribute dominantly to testicular size (26, 27). Congenital cryptorchidism is associated with low birth weight (28) and reduced post-pubertal testicular size, most likely reflecting a small number of Sertoli cells (27, 29). Forty percent of males with SRS present with congenital malformations of the external genitals, indicating disturbed androgen action supposedly due to abnormal gonadal function during fetal life (1, 30).

AMH secreted by immature Sertoli cells, reliably reflects the function of testes in prepubertal boys, and consequently, low serum AMH is found to correlate with small testes and decreased amounts of functional Sertoli cells (31, 32). In this study, a number of NRs had subnormal AMH concentrations prepubertally. With the onset of puberty and with increasing concentrations of T, AMH naturally declines as Sertoli cells mature (33). Despite the lack of difference in the age of pubertal onset (5), NRs had a natural drop in AMH concentrations 2 years earlier than did Rs, indicating a disturbed testicular function. Furthermore, at 12 years of age, AMH was positively correlated with AH outcome, and AMH concentrations were significantly lower in NRs than in Rs, indicating a different timing of secretion between the groups. The AMH secretion patterns of NRs and Rs may be explained by either excessive exposure to T in NRs, a pathological small number of Sertoli cells or may suggest a combination of both.

Inhibin B is also produced by Sertoli cells (34, 35) and serves as a sensitive marker of Sertoli cell damage after puberty but remains germ cell independent before puberty (36, 37). In a previous report, based on pubertal and post-pubertal inhibin B concentrations below reference intervals, Sertoli cell dysfunction was suspected to be common in males with SRS (7). In the present study, possibly due to the small number of patients, we found no correlation with AH outcome or differences in inhibin B concentrations between the groups at any age, and the majority of samplings were within previously reported normal reference intervals (37, 38). In summary, we found AMH secretion patterns but not inhibin B secretion patterns indicating Sertoli cell dysfunction. It is possible that an imbalance between T secreting Leydig cells (34, 35) and Sertoli cells may explain the findings of androgen secretion patterns.

Unique to this study is the longitudinal design with few missing data. Furthermore, sex steroid concentrations were analyzed using mass spectrometry-based methods, offering more reliable results. The major weakness of the study is the limited number of patients, which might have influenced the results, and studies of larger cohorts and with long follow-up periods are needed to confirm the results of this study.

In conclusion, this study shows a negative correlation between DHEAS from the age of 8 years as well as other androgens from 10 years of age and AH outcome consistent with higher concentrations of androgens in NRs than in Rs from the age of 10 years. The hypothesis of this study, that testicular activity rather than adrenal activity causes increased androgen secretion, leading to elevated E_2_ concentrations through aromatization in GH-treated males with SRS is rejected, as the findings of this study suggest that dysfunction of both the adrenal and gonadal glands result in increased androgen secretion that may be present in a substantial number of males with SR. The importance of investigating androgen concentrations with a reliable method during childhood and puberty to identify patients at risk of impaired AH outcome should not be underestimated.

## Data Availability Statement

The datasets generated for this study will not be made publicly available due to privacy policy. Requests to access the datasets should be directed to KK, kjersti.kvernebo.sunnergren@gu.se.

## Ethics Statement

This study was carried out in accordance with recommendations of the Regional Ethical Review Board in Gothenburg (449-16), who approved the protocol. Written and informed consent were obtained from the parents and retrospectively from the patients in accordance with the Declaration of Helsinki.

## Author Contributions

JD was responsible for care and treatment of the patients. KK, CA-L, and JD contributed to the study design, interpretation, and analysis of data. CA-L was responsible for the mass spectrometry analysis. KK wrote the first draft of the manuscript. CA-L and JD contributed to the final writing and revising of the manuscript, and checked for important intellectual content. All authors approved of the final manuscript as submitted.

### Conflict of Interest

JD received unrestricted grants from Pfizer. The remaining authors declare that the research was conducted in the absence of any commercial or financial relationships that could be construed as a potential conflict of interest.
