# Supplementary material for: Adrenal and Gonadal Activity, Androgen Concentrations, and Adult Height Outcomes in Boys With Silver-Russell Syndrome
Source: Front Endocrinol (Lausanne). 2019 Dec 10;10:829. doi: 10.3389/fendo.2019.00829 (PMC6914679; doi:10.3389/fendo.2019.00829)
Supplement: Supplementary file 1 [file Table_1.docx]

**Supplement:** Demographic description of hormone analyses, expressed as median (range), for eight non-responders and five responders

| Age (Years) | 6 | | 8 | | 10 | | 12 | | 14 | | 16 | |
| --- | --- | --- | --- | --- | --- | --- | --- | --- | --- | --- | --- | --- |
| NR/R | NR | R | NR | R | NR | R | NR | R | NR | R | NR | R |
| DHEAS (μmol/L) | 0.7  (0.1-1.5) | 0.3  (0.1-0.9) | 1.9  (0.4-4.0) | 0.5  (0.2-1.7) | 2.9  (1.1-5.4) | 1.0 ^*^  (0.6-2.9) | 3.7  (1.9-7.7) | 2.2  (0.9-5.1) | 4.4  (2.3-9.5) | 2.9  (1.1-5.9) | 5.0  (2.8-8.0)^2^ | 4.5  (2.0-7.4)^1^ |
| A_4_ (nmol/L) | 0.4  (0.2-1.1) | 0.3  (0.2-0.5) | 0.7  (0.4-1.5) | 0.5  (0.2-1.1) | 1.1 ^*^  (0.9-1.4) | 0.6 ^*^  (0.4-1.1) | 1.7 ^*^  (1.2-3.2) | 0.8 ^*^  (0.7-1.5) | 2.5  (1.9-3.9) | 1.5  (0.8-2.9) | 2.2  (1.2-3.4)^2^ | 2.1  (1.4-3.7)^1^ |
| T (nmol/L) | 0.2  (<0.1-0.3) | 0.1  (<0.1-0.2) | 0.2  (0.1-0.3) | 0.2  (0.1-0.3) | 0.3  (0.3-0.8) | 0.1 ^**^ (0.1-0.1) | 7.8  (0.4-11.8) | 0.2 ^**^  (0.1-0.3) | 15.6  (12.2-20.2) | 10.4 ^*^  (0.2-14.9) | 17.1  (9.0-21.8)^2^ | 16.0  (1-19.0)^1^ |
| DHT (pmol/L) | 41  (<27-84) | <27  (<27) | 50  (<27-91) | <27  (<27-55) | 122  (51-146) | 28 ^**^ (<27-66) | 652  (64-1191) | 59 ^**^  (<27-85) | 1137  (781-1493) | 872  (67-1671) | 1130  (538-1304)^2^ | 838  (171-1169)^1^ |
| SHBG (nmol/L) | 76  (44-116)^1^ | 61  (27-120) | 62  (30-184) | 66  (37-133) | 52  (35-164) | 51  (37-130) | 41  (18-157) | 38  (35-128) | 27  (20-63)^1^ | 43  (23-103) | 29  (17-58)^2^ | 26  (13-45)^1^ |
| FAI T  (T/SHBG)  x10^4^ | 15  (5-59)^1^ | 28  (4-44) | 41  (10-63) | 27  (14-38) | 59  (44-112) | 26 ^**^  (10-38) | 1536  (91-4609) | 43 ^**^  (17-74) | 6325  (3211-8923)^1^ | 2214 ^*^  (52-5707) | 5491  (3761-  6811)^2^ | 7120 (227-14124)^1^ |
| FAI DHT  (DHT/SHBG)x10^4^ | 3  (1-12)^1^ | 2  (1-5) | 8  (1-27) | 3  (2-7) | 23  (3-38) | 5 ^*^  (1-13) | 132  (15-331) | 16 ^*^  (4-21) | 374  (189-679)^1^ | 208  (16-379) | 364  (200-483)^2^ | 437  (38-655)^1^ |
| AMH (ng/mL) | 64  (30-125)^3^ | 90  (54-147) | 51  (30-126) | 75  (55-137) | 42  (23-108) | 59  (37-108) | 11  (4-35)^3^ | 50 ^**^  (18-134) | 6  (3-9) | 8  (2-31) | 6  (4-21)^2^ | 6  (2-13)^1^ |
| Inhibin B (pg/mL) | 93  (60-128)^3^ | 73  (57-181) | 72  (64-134) | 91  (49-171) | 97  (59-207) | 97  (31-142) | 160  (69-382)^3^ | 142  (43-212) | 135  (111-274) | 168  (62-329) | 168  (119-280)^2^ | 150 (131-261)^1^ |

^*^ *P*-values <0.05, ^**^ *P*-values <0.01, Mann-Whitney U test was used for statistical analysis.

^1^ One missing data

^2^ Two missing data

^3^ One extrapolated value

**Abbreviations**

A_4_=Androstenedione

AMH=Anti-Müllerian hormone

DHEAS=Dehydroepiandrosterone-sulphate

DHT=Dihydrotestosterone

FAI=Free androgen index

NR=non-responder

R=responder

SHBG=Sex hormone binding globulin

T=Testosterone
